# Preparation and Application of Degradable Lignin/Poly (Vinyl Alcohol) Polymers as Urea Slow-Release Coating Materials

**DOI:** 10.3390/molecules29081699

**Published:** 2024-04-09

**Authors:** Yue Liu, Long Cao, Linshan Wang, Yanjiao Qi, Yamin Zhao, Huining Lu, Lina Lu, Derong Zhang, Zifan Wang, Hong Zhang

**Affiliations:** 1 China-Malaysia National Joint Laboratory, Biomedical Research Center, Northwest MinZu University, Lanzhou 730000, China; y211830601@stu.xbmu.edu.cn (Y.L.); y221830804@stu.xbmu.edu.cn (L.C.); 1zz.316@163.com (D.Z.); xbmuswyx@163.com (Z.W.); 2 Key Laboratory for Utility of Environment-Friendly Composite Materials and Biomass, Universities of Gansu Province, Lanzhou 730000, China; 280112007@xbmu.edu.cn (Y.Z.); 283091685@xbmu.edu.cn (L.L.); 3 Key Laboratory of Environment-Friendly Composite Materials of the State Ethnic Affairs Commission, Lanzhou 730000, China; y231830857@stu.xbmu.edu.cn (L.W.); gszhangh@126.com (H.Z.); 4 Gansu Provincial Biomass Function Composites Engineering Research Center, Lanzhou 730000, China; 179071486@xbmu.edu.cn; 5 Department of Life Sciences and Biological Engineering, Northwest University for Nationalities, Lanzhou 730124, China

**Keywords:** lignin, slow-release, degradation, dynamics model, plant growth

## Abstract

The massive amount of water-soluble urea used leads to nutrient loss and environmental pollution in both water and soil. The aim of this study was to develop a novel lignin-based slow-release envelope material that has essential nitrogen and sulfur elements for plants. After the amination reaction with a hydrolysate of yak hair keratin, the coating formulation was obtained by adding different loadings (2, 5, 8, 14 wt%) of aminated lignin (AL) to 5% polyvinyl alcohol (PVA) solution. These formulations were cast into films and characterized for their structure, thermal stability, and mechanical and physicochemical properties. The results showed that the PVA-AL (8%) formulation had good physical and chemical properties in terms of water absorption and mechanical properties, and it showed good degradation in soil with 51% weight loss after 45 days. It is suitable for use as a coating material for fertilizers. Through high-pressure spraying technology, enveloped urea particles with a PVA-AL (8%) solution were obtained, which showed good morphology and slow-release performance. Compared with urea, the highest urea release was only 96.4% after 30 days, conforming to Higuchi model, Ritger–Peppas model, and second-order dynamic model. The continuous nitrogen supply of PVA-AL coated urea to *Brassica napus* was verified by potting experiments. Therefore, the lignin-based composite can be used as a coating material to produce a new slow-release nitrogen fertilizer for sustainable crop production.

## 1. Introduction

Fertilizers have been used to increase crop yields for more than 140 years. Due to their high nitrogen content (46%), the most commonly used urea fertilizers can help plants to synthesize proteins and chlorophyll [1]. However, traditional fertilizers, especially nitrogen fertilizers, have a serious nutrient loss problem when applied to cultivated land, and the average utilization rate is only about 30–35%. The loss of fertilizer efficiency is due to the high leaching or instantaneous release caused by high solubility as well as partly to the volatilization loss of N [2]. Finding ways to effectively avoid the rapid leaching of urea nitrogen fertilizer is the current problem. By introducing slow-release agents to control the release rate, slow-release fertilizers can prolong the release time of the fertilizer, making it release slowly and keep pace with the nutritional needs of plants and thereby increasing the utilization rate. At the same time, slow-release fertilizers reduce environmental pollution such as soil degradation and water eutrophication [3]. Current solutions, such as coated urea [4,5], hybrid microspheres [2], and hydrogel-doped urea, are among the more common methods of handling urea developed in recent years [6]. However, the process of mixed microspheres and hydrogels are complex and costly, which seriously reduces the economic benefits of slow-release fertilizers. Film-coated fertilizers are formed by wrapping a layer of inorganic or organic substances, such as sulfur-coated fertilizers (SCU) or polymer-coated fertilizers (PCU), on the surface of urea [3]. However, the sulfur material itself does not have good deformability and the SCU is fragile, which may lead to cracking and breaking of the coating [7]. Thus, the release of SCU is not very controllable and there is no way to achieve a stable and uniform release. It has been reported that PCU and SCU can increase rice yield by 8.6 and 1.2%, respectively [8]. In addition, nitrogen release from SCU can led to a reduction in the number of effective spikes at maturity and a reduction in the total number of spikes, ultimately leading to significantly lower yields than control [3]. Therefore, common polymer polymers are often used to make coated urea. For example, Jinta et al. investigated the feasibility of coating urea particles with polylactic acid–ethyl phthalate materials [9]. Mathews et al. used the poly n-isopropylacrylamide (PNIPAm-PU) copolyurethane (PNIPAm-PU) coating method to prepare urea particles [10]. However, there are many drawbacks, such as the complexity of the manufacturing process, the requirement for costly chemicals, and serious environmental challenges during recycling and disposal. In addition, certain polymer coatings are not easily degradable and may affect the ecological cycle [11]. Therefore, polyvinyl alcohol was selected as the main raw material to prepare slow-release coating, which has a simple structure and is easily degradable [12]. However, the mechanical properties, compression resistance, and water resistance of polyvinyl alcohol alone are not ideal [13]. A number of methods for modifying the polyvinyl alcohol membrane have been derived. 

After the doping of PVA in the nanocellulose extracted from wheat straw, the mechanical properties and antioxidant capacity of the whole membrane were significantly improved [14]. Nguyen et al. crosslinked chitosan and PVA blends with urea as the base material while using glutaraldehyde as the cross-linking agent, and the formulation enabled the extended release of urea up to 10 d, which is a potential slow-release fertilizer for agriculture [6]. Mittal et al. [12] prepared polyvinyl alcohol (PVA)/starch (St) co-blended membranes, urea-formaldehyde crosslinked PVA/St membranes, and composite membranes of palmitic acid grafted copolymerized barley husk (BH) and grafted BH, respectively. The tensile strength of the grafted BH composite film was 72.4% higher than that of the PVA/St composite film degradable clay–polymer (starch/poly(vinyl alcohol)) co-encapsulated blended membranes (CPSBs) prepared by Sarkar, Abhijit [15]. This was then used to produce encapsulated diethylammonium phosphate (DAP), which reduced the release of N and P significantly. Li et al. [16] prepared a slow-release fertilizer bilayer coating material using low-cost poly(vinyl alcohol) (PVA), with methyl cellulose (MC) as the inner coating material and bumpy clay doped into the highly absorbent polymer poly(acrylic acid) (PAA) along with poly(acrylamide) grafted with natural biodegradable lignin (PAM) as the outer coating material. The results indicated that the cumulative release of urea molecules from this coating material was 85.10%. Elhassani et al. extended the release of the coated urea up to 18 days by mixing chitosan (Cs) and polyvinyl alcohol (PVA) followed by the addition of lignin nanoparticles (LNPs) with different loadings (1, 2, 3, and 5 wt% LNPs) [17].

At present, biopolymers and other coating materials are always used to prepare slow-release fertilizers; however, they are either too cumbersome in process or have poor slow-release properties. Therefore, in this study we aimed to develop a new simple, inexpensive, and well-performing membrane-coated slow-release material. Yak hair keratin-aminated lignin was doped into PVA for cross-linking, then the urea was coated by spraying with a round drum granulator. The release behavior of different levels of membrane-coated urea and the biodegradation performance in the soil were investigated as well. Finally, the effect of the coated urea on the growth and development of *Brassica napus* was studied. 

## 2. Characterization

### 2.1. Characterization of L, AL and PL Composite Films

**Figure 1:**
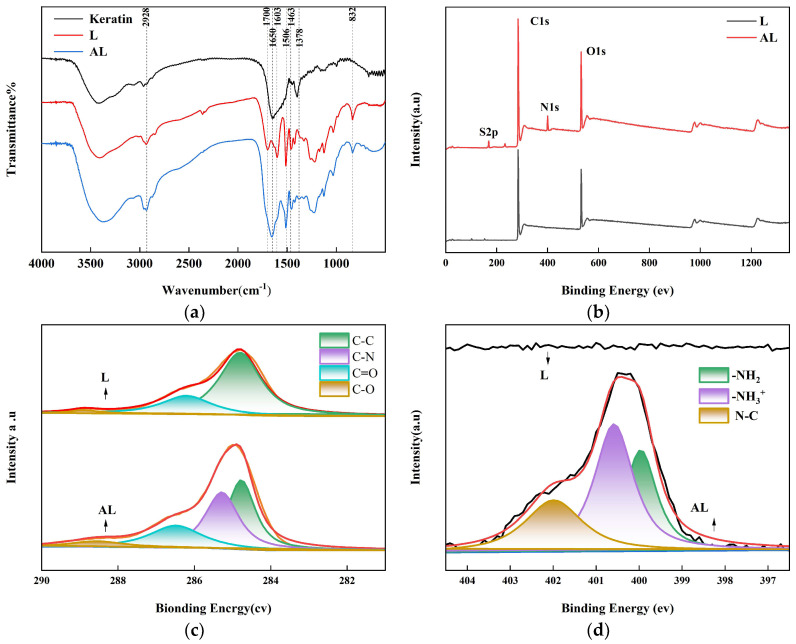
FTIR spectrum of the keratin L, AL (**a**), XPS spectra of L, AL (**b**), high-resolution image of C1 of L, AL (**c**), and high-resolution image of N1 of L, AL (**d**).

The FTIR spectra of the aminated lignin and lignin (Figure 1a) clearly reflected the structure differences. The broad peak at 3404 cm^−1^ was assigned to the hydroxyl groups in the aliphatic and phenolic structures of the lignin [18]. The C-H asymmetrical (2928 cm^−1^) and symmetric stretching (2840 cm^−1^) vibrations in the methyl and methylene structures were always observed. Furthermore, the peaks at 1603 cm^−1^ and 1506 cm^−1^ were derived from the tensile vibration of the aromatic skeleton, and the peaks of 1463 cm^−1^ and 832 cm^−1^ are attributed to the C-H bending and out-of-plane deformation vibrations, respectively [18]. It was suggested that the backbone structure of lignin was not disrupted during the Mannich reaction, such as guaiacyl (1260 cm^−1^), syringyl structures (1220 cm^−1^), and ether bond of the lignin structure (1123 cm^−1^), which can also be observed in the spectrum. In addition, the C-H vibration peak intensity of the lignin aromatic skeleton was significantly reduced in the AL spectrum, such as 1603 cm^−1^, 1506 cm^−1^, 1463 cm^−1^, and 832 cm^−1^, suggesting that the Mannich reaction occurs primarily in the phenolic hydroxyl group [19]. In contrast, some peak intensities were greatly increased after the Mannich reaction, such as C-H stretching vibration in the methyl structure at 2928 cm^−1^. This may due to the successful introduction of keratin during the Mannich reaction [18,20]. The formation of the aminated lignin was also confirmed by the peak centered on 1378 cm^−1^, 1650 cm^−1^, and 1378 cm^−1^, which arose from the stretching vibration of bonding C-N [21,22,23].

XPS spectra of AL further confirmed that the yak keratin hydrolysate was successfully grafted onto the molecular chain of alkaline lignin (Figure 1b). Compared with the characteristic peaks of carbon (C1s, 284.8 eV) and oxygen (O1s, 532 eV) of the L, there were more extra peaks of nitrogen (N1s, 399.8 eV) on the AL sample (Figure 1b,d), which further confirmed the success of the graft reaction [24,25]. The generated sulfur peak (S2p, 289.2 eV) was mainly ascribed to the sulfur-containing amino acid of the yak hair keratin, such as cysteine, which was further evidenced by the LC-MS/MS analysis of the hydrolysate (Figure S1, Table S1). The high-resolution C1s of AL also showed a new peak at 285.3 eV, as shown in (Figure 1c), which belongs to the C-N group formed by amination of lignin. 

**Figure 2:**
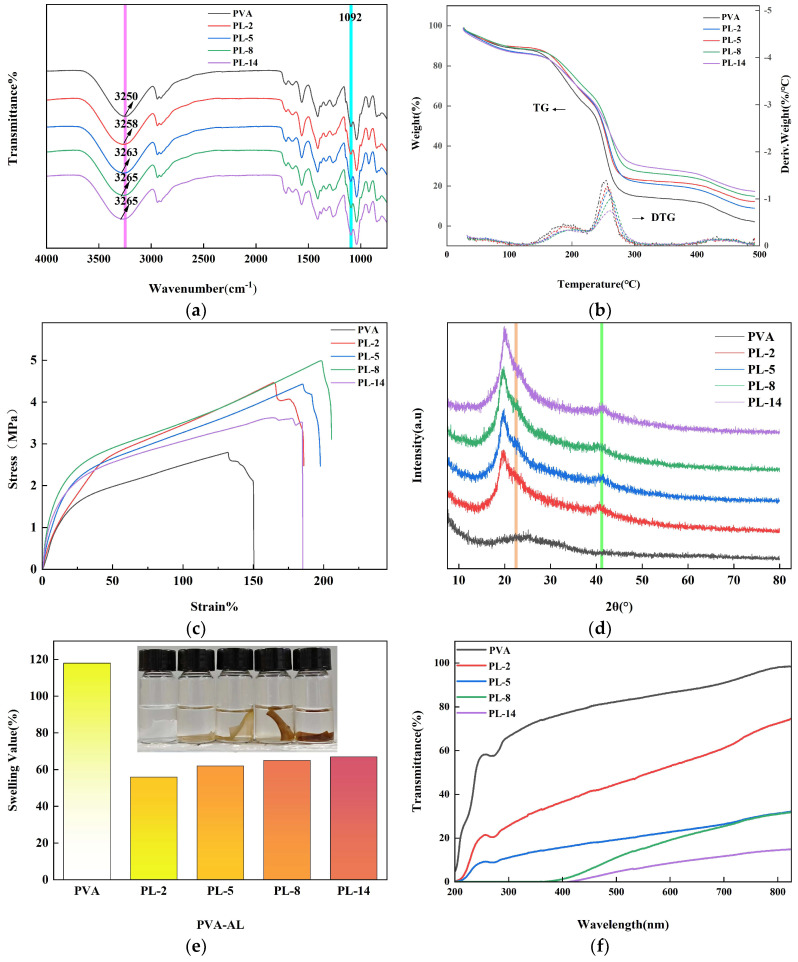
Infrared spectra (**a**), thermogravimetric (**b**), stress–strain (**c**), XRD (**d**), solubility (**e**), and UV optical resistance (**f**) of PVA, PL-2, PL-5, PL-8, and PL-14.

FTIR spectra were used to analyze the blending mechanism of AL with PVA by analyzing the change in the position of the absorption peaks after mixing. As shown in Figure 2a, the FTIR spectra of the pure PVA film and the individual PL nanocomposite films are highly similar. All absorption peaks were PVA [26], but no characteristic peaks of lignin were found to appear in PVA, which might be due to too little addition. Of lignin In addition, the C-O stretching vibrational absorption peak at 1092 cm^−1^ changed slightly with the introduction of aminated lignin. The pure PVA films exhibited typical tensile vibrations of hydroxyl groups (O-H) centered at 3250 cm^−1^ with the increased content of LNSs. The original PVA showed a gradual blue shift at 3250 cm^−1^ in the PL, and the absorption peak of (O-H) shifted from 3250 cm^−1^ to 3265 cm^−1^ in the composite film, which is due to the formation of strong hydrogen bonds between lignin and PVA [27,28,29,30]. Therefore, the addition of aminated lignin affected the overall structure of PVA. FTIR also suggested that PVA and AL were mainly mediated through physical mixing rather than chemical reactions [26].

The TG/DTG curves of pure PVA and PL composite membranes are shown in Figure 2b. It can be seen that the weight loss at 25–150 °C is the result of the loss of water and glycerol in the membrane; the weight loss at 150–300 °C is related to the chain breakage and thermal degradation of the PVA side chains, whereas the weight loss at 400–500 °C is related to the thermal decomposition of the PVA main chains. With increasing content of the aminated lignin, the temperature at the maximum thermal decomposition rate of the PL composites increased from 249.8 °C for the original PVA to 261.7 °C for PL-14. This could be attributed to the strong intermolecular hydrogen bonding interactions between the PVA matrix and the aminated lignin. The aromatic structural units in the aminated lignin molecule also play an important role in improving the thermal stability [31]. Because AL plays an important role in improving the thermal stability of PVA nanocomposite films, it provides a meaningful way to achieve high-performance PVA composites. 

The hydrogen bonding between PVA and aminated lignin was further verified by X-ray diffraction (XRD). As shown in Figure 2d, the diffractogram of PL has a peak at 2θ = 19.4° and a shoulder at 2θ = 22.5°, which corresponds to the characteristic signals of semi-crystalline PVA [32]. The intensities of these two diffraction signals are almost unchanged in all PL membranes. A new weak peak of diffraction at 2θ = 40.2° appears in the PL composite film compared to the pure PVA film. Using Bragg’s equation (d = λ/(2sinθ), λ = 0.154 nm, 2θ = 40.2°), the distance between the chains of aminated lignin and PVA molecules was determined to be 0.224 nm, which is consistent with the hydrogen bonding interaction distance of 0.225 nm. This further confirms the hydrogen bonding interaction between the aminated lignin and the PVA matrix. This is consistent with the findings of the FTIR analysis [33]. 

### 2.2. Properties of Membrane and Degradation 

#### 2.2.1. Stress–Strain of the Films

The tensile properties of PVA/PL films were investigated by tensile tests. Figure 2c shows the effect of the blending ratio on the tensile strength and break elongation of PVA/aminated lignin blended films. The tensile strength and break elongation of pure PVA films were 150.65% and 2.79 MPa, respectively. It can be seen from the figure that the tensile strength of PVA/amidated lignin films increased and then decreased with the increase of lignin dosage. The results show that the incorporation of aminated lignin increased the tensile strength and elastic modulus of PVA; this phenomenon can be explained by the generation of strong hydrogen bonding between aminated lignin and PVA [34,35,36]. In addition, due to its rigid structural component, aminated lignin can be used as a crosslinker in linearly structured polyvinyl alcohols [37]. The break elongation of PL film showed an increasing and then decreasing trend with the increased addition of aminated lignin, and the growth of tensile strength also stagnated. This is due to the network structure formed by aminated lignin and PVA, which enhances the performance of the whole PVA film [38,39]. Because the hydrogen bonds formed between aminated lignin and PVA are gradually saturated, the excess unreacted aminated lignin is doped in the membrane; consequently, the performance of the membrane is affected [28]. 

#### 2.2.2. Water Absorption

Water swelling is one of the most significant drawbacks of biopolymers in wrap material applications [40]. Figure 2e shows the swelling rate of the membrane after the swelling experiment. The results show that after reaching the swelling equilibrium, there is a significant increase in volume and mass of pure PVA films and all PVA/AL films. Among all the tested films, PVA film showed the highest swelling rate of about 118 ± 1.32%, which may be attributed to the hydrogen bonding between aminated lignin and PVA forming a denser network. In addition, the water swelling rate of the PVA/AL film was significantly reduced. The water absorption of PL, however, increased slightly with the increase in aminated lignin content, which might be due to the failure of cross-linking of aminated lignin to increase the swelling of the composite film [27]. 

#### 2.2.3. UV Shielding Performance

Figure 2f shows the UV–visible transmission spectra of pure PVA membrane, PVA co-blended membrane, and PVA nanocomposite membrane, all with thicknesses of around 100 μm and with different AL contents. Compared with pure PVA membrane, which hardly affects the UV transmittance, the composite membrane shows stronger absorption in the UV spectrum, which may be due to the presence of AL [41]. It was evident that the UV–visible transmittance of the films decreased with increased AL content. The UV transmittance of pure poly (vinyl alcohol) film was 69.61%, whereas the UV transmittance of composite films PL-2, PL-5, PL-8, and PL-14 was 29.25%, 12.64%, 0.13%, and 0%, respectively. This is due to the fact that the PVA nanocomposite films contain high content of LNS, which makes them more absorbent to UV–visible light [42]. The ability of lignin incorporation to provide UV shielding is mainly dependent on the large number of color rendering groups in lignin, such as phenolic hydroxyl groups, methoxy groups, and benzene rings [43,44,45,46]. In addition, the proportion of the dispersed phase in the composite film increased, which may lead to higher refraction and reflection of light [47].

#### 2.2.4. The Morphology Structure and Degradation Properties 

**Figure 3:**
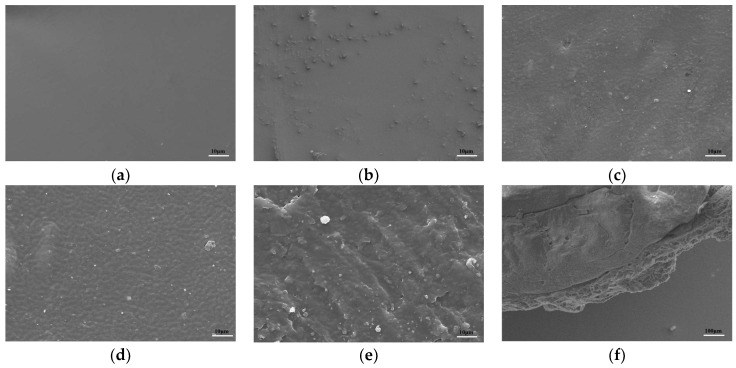
SEM images of cross-sections of PVA (**a**), PL-2 (**b**), PL-5 (**c**), PL-8 (**d**), PL-14 (**e**), and UPL-2 (**f**) slices.

The surface morphology of the pure PVA and composite membranes were characterized as shown in Figure 3a–e. It was showed that the surface of the PVA membranes had a homogeneous and smooth structure. There was no large-scale agglomeration in PL-2, PL-5, or PL-8, indicating that the AL particles were well dispersed in the PVA solution [27]. On the contrary, PL-14 showed a large number of irregular AL clusters under SEM. This large-scale agglomeration may be caused by the incomplete dissolution of excessive AL content. It can be seen from the infrared, stress–strain, and water absorption properties that the performance of PL-14 is affected by this excessive AL agglomeration. Because its performance is remarkable in many aspects with respect to the control groups, PL-8 was selected as the spraying reagent for use in further studies. 

**Figure 4:**
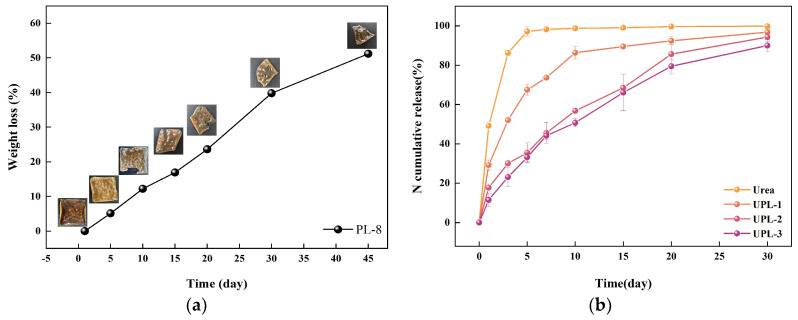
Soil degradation capacity of membrane PL-8 (**a**) and 30 day soil retardation capacity of urea, coated urea UPL-1, UPL-2, and UPL-3 (**b**).

The degradation property of coating materials is an important parameter for designing coated fertilizers for agricultural applications with less negative impact on the environment [48]. We investigated the weight loss (%) of the film after burying it in moist soil for 45 days (Figure 4a). The results showed that the size of the film decreased and the surface of the film became rough and wrinkled after burial in soil. The degradation rate of PL membranes was 16.9% after 15 days of burial in soil, with 51% weight loss observed after 45 days. This is approximately the same as other PVA-doped biomass material films [49]. This was mainly ascribed to the water absorbed by the film expanding the film and accelerating the growth of microorganisms on the surface, promoting the degradation of these composite membranes [12,50]. The above results showed that PL has good biodegradability in soil environments.

### 2.3. The Sustained-Release Properties of Coated Urea

The coated urea granules were obtained after a spraying operation under high speed rotation. It can be seen from Figure 3f that the urea particles were wrapped with a uniform composite film outside; the SEM image of the cross-section shows that the coating has an obvious hierarchical structure. This is due to the evaporation of the coating solvent that prevents the coating layer from being fully adhered. When immersed in water, these hollow layers are preferentially filled, which to some extent slows down the entry rate of water into the fertilizer particles. A soil column leaching experiment was carried out to analyze the slow-release properties. It can be seen from Figure 4b that the release of urea from the uncoated film was relatively slow within first 5 days. This was mainly due to the fact that the hydrophobic membrane material could inhibit the dissolution of urea to some extent. From 5 to 10 days, the rate of urea release increased significantly, and the film material began to swell due to water absorption and the coinstantaneous leaching of urea. [51,52]. At the same time, the film material was degraded to a certain extent, resulting in rapid release of urea. The release rates reached a plateau at days 10 and 30, respectively, which may be attributed to the formation of hydrogen bonds and a three-dimensional mesh structure between the aminated lignin and PVA membranes, which slowed down the cumulative release rate of urea. In the present study, the urea release rates of urea pellet, membrane-coated UPL-1, UPL-2, and UPL-3 reached 99.8%, 96.46%, 94.33%, and 90.16%, respectively, after 30 days 

**Figure 5:**
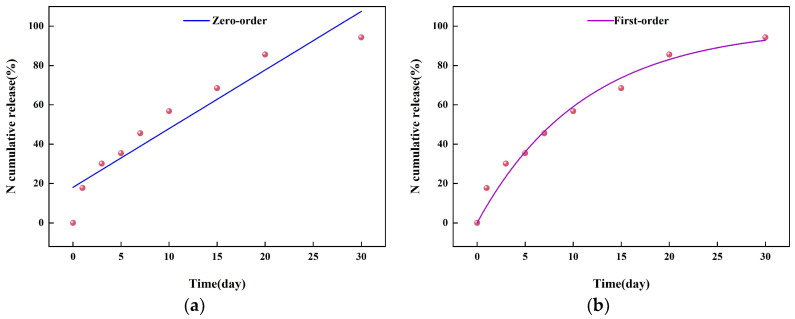

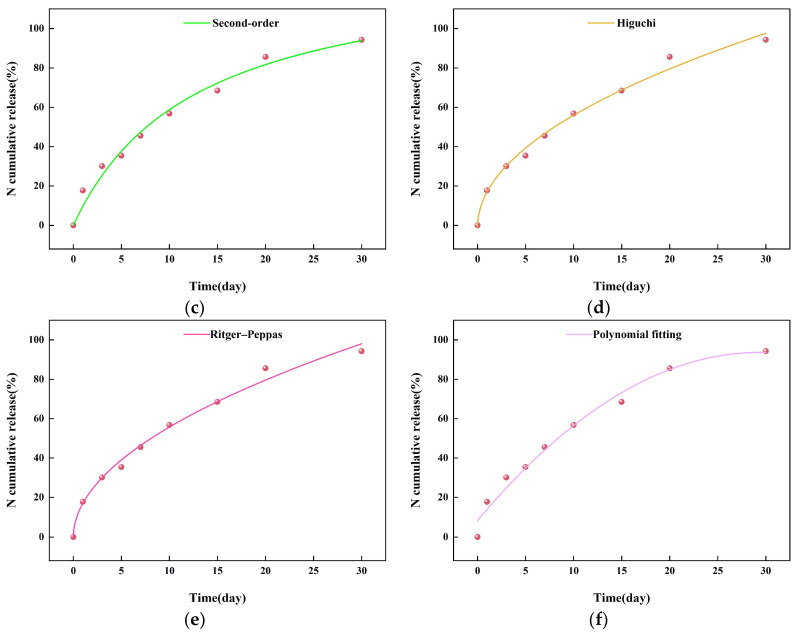
Kinetic modeling of UPL-2 in soil: zero-order kinetic model (**a**), first-order kinetic model (**b**), second-order kinetic model (**c**), Higuchi model (**d**), Ritger-Peppas model (**e**), and polynomial fitting model (**f**).

Based on these results, the PL-coated materials exhibited slow-release properties comparable to results reporting that the release of K, P, and N were 96.23%, 95.48%, and 96.31%, respectively, after PVA@CNC14.5%-c-NPK coating product was released in soil for one month [53]. This indicates that AL/PVA film has a slow-release effect with respect to urea and can be used as a coating material with slow-release properties. The results show that the thickness of the coating is an important parameter that can significantly control nutrient release [54]. In addition, based on the experimental data on urea release from the composite membranes, different kinetic models were used to fit the retardation curves of urea. The fitting results are shown in Table 1 and Figure 5. The coefficient of determination R^2^ was used to evaluate the degree of fitting. From the fitting equations and the coefficient of determination R^2^, the validity of the fitted retardation models was in the following order: Ritger–Peppas model > Higuchi model > second-order kinetic model > polynomial fitting model > first-order kinetic model > zero-order kinetic model.

Analysis of the results in Table 1 shows that the in vitro release profiles of the formulations fit well with the Ritger–Peppas equation. With n describing the release mechanism [48], when *n* ≤ 0.45, the drug was released mainly by the Fick diffusion mechanism; when 0.45 < *n* < 0.89, urea was released by diffusion and solubilization in a coexisting manner; when *n* > 0.89, the drug was released mainly by solubilization; and when *n* > 0.89, the drug was mainly released by solubilization. Therefore, in this text, *n* (0.53359) was located in the range of 0.45–0.89, indicating that urea was released by both diffusion and solubilization.

**Figure 6:**
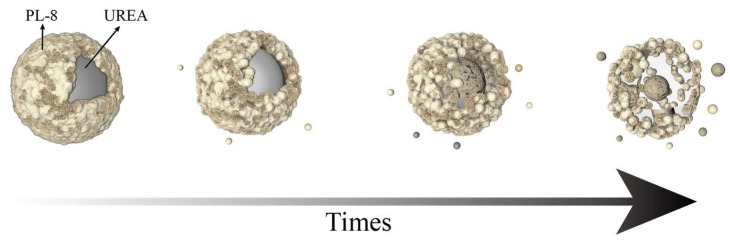
Soil slow-release mechanism of encapsulated urea UPL.

Therefore, it is suggested that the urea release mechanism of AL/PVA film is as follows (Figure 6). First, the water molecules come into contact with the polymer film and are slowly absorbed by the film. Water molecules are immediately dissolved on contact with urea through dynamic spatial migration with the polymer film macromolecules [55]. the film absorbs the water in the soil and starts to dissolve, and the denseness of the film decreases. It is believed that the degree of denseness of the film affects the rate of water evaporation, as well as the rate of evaporation; the lower the degree of denseness of the film, the faster the water diffuses into the film. On the contrary, the structure of AL/PVA film becomes denser, and can effectively block the water. Therefore, film that is cross-linked with aminated lignin plays an important role in slowing the release rate, as verified by our swelling experiment. Second, the surface of the film is dissolved, then the urea inside the material dissolves and spreads with the dissolution and diffusion of the AL/PVA matrix.

The release performance of the film urea may additionally be related to the degradation of the film material in the soil; however, there are fewer studies in this area [56]. Figure 4a summarizes that the film continued to show a good degradation effect as the number of days increased. According to our degradation experiment, the envelope material had been degraded by 5.1% on the fifth day; thus, the envelope urea was released to a certain extent at the beginning. This means that the film degradation and urea leaching processes are simultaneous, which is very close to the Ritger–Peppas slow-release model during the soil column leaching experiment. From this, it can be deduced that the degradation of the film is closely related to the slow-release property of the encapsulated urea.

### 2.4. Potting Experiments

**Figure 7:**
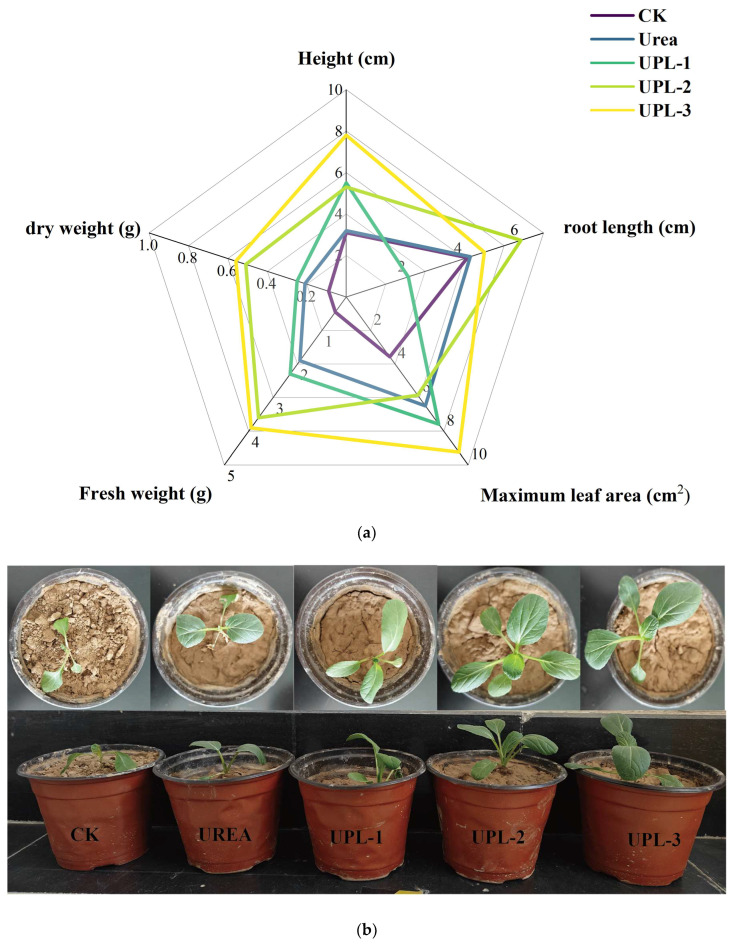
Effects of different fertilizers on plant growth of *Brassica napus*: plant height, root length, maximum leaf area, fresh weight, and dry weight (**a**) and the state of the plant growth (**b**).

To further verify the effect of UPL on plant growth, UPL-1, UPL-2, and UPL-3 were used to treat *canola* potting trials. Plant growth trials were evaluated by measuring changes in plant height, root length maximum leaf area, fresh weight, and dry weight. As can be seen from Figure 7, UPL-2 and UPL-3 had significant promotional effects on plant height, root length maximum leaf area, fresh weight, and dry weight of *canola* plants. Overall, the plant height was relatively low; this was probably due to the cold western weather, poor soil, and limited growth time during the experiment. The plant height of the UPL-3 application was 2.5 cm more than UPL-2 and 4.7 cm higher than the control. For the maximum leaf area and fresh weight, UPL-3 reached a very significant level in the wrapped urea treatment. Moreover, the root length of UPL-3 was 1.3 cm shorter compared to UPL-2, which may be due to root breakage caused by mistake during the removal of plants from soil for treatment. The importance of fertilizer efficiency for plant growth is evident from the significant increase in the dry weight of plants in several sets of urea-treated experiments. Compared with other treatments, the promotional effects of UPL-2 and UPL-1 were not significant, which was probably due to the incomplete encapsulation of the urea and the thin covering layer, leading to rapid dissolution of the partial urea. This is further verified by the data from the soil column leaching experiment.

## 3. Methods

### 3.1. Materials

Corn cob industrial lignin (L) was supplied by Shandong Longlive Bio-technology Co., Ltd., China; the total lignin content was higher than 94%. Hydrochloric acid (HCl), sodium hydroxide (NaOH), glutaraldehyde, propanetriol solution, and polyvinyl alcohol (PVA) used in this work were purchased from Shanghai Aladdin biochemical technology Co., LTD, China. Yak hair (keratin content 81.9%, fat content 4.85%, and ash content 4.92%) was obtained from the black yaks on the western plateau of China. Other reagents were analytically pure and used without further treatment.

### 3.2. Liquid Chromatography (LC)–Electrospray Ionization (ESI) Tandem MS(MS/MS) Analysis

According to the universal sample preparation method [57], 50 μg samples were dissolved in 1 mL 0.1% trifluoroacetic acid and centrifugally ultrafiltered with a 10 kd ultrafiltration tube (pall OD010C35) at 12,000× *g*. The hydrolysate solution was desalted on C18 Cartridges, then concentrated by vacuum centrifugation and reconstituted in 40 µL of 0.1% (*v*/*v*) formic acid. All samples were stored at −80 °C until LC-MS/MS analysis. Samples (5 μg) were loaded onto a C18-reversed phase column (Thermo Scientific Easy Column Waltham, MA, USA, 10 cm long, 75 μm inner diameter, 3 μm resin) in buffer A (2% acetonitrile and 0.1% Formic acid) and separated with a linear gradient of buffer B (80% acetonitrile and 0.1% formic acid) at a flow rate of 250 nL/min controlled by IntelliFlow technology over 60 min. The dynamic exclusion duration was 25 s. Survey scans were acquired at a resolution of 70,000 at *m*/*z* 200 and the resolution for HCD spectra was set to 17,500 at *m*/*z* 200. The MS data were analyzed using MaxQuant software version 2.1.0.5. MS data were searched against the UniProtKB Bos mutus database.

### 3.3. Preparation of Aminated Lignin

First, 3 g of yak hair, 5% KOH, and 40 mL deionized water were added to a 100 mL flask and stirred constantly. After stirring for 7 h at 70 °C, the solution was filtered to remove the impurities. The keratin hydrolysate was finally obtained after dialysis and cryodesiccation. The amination of the lignin was carried out under alkaline condition by Mannich reaction. Specifically, 1 g L was first dissolved in 10 mL 0.4 mol/L sodium hydroxide solution and stirred continuously for about 15 min to ensure sufficient dissolution. Then, a certain amount of amination reagent, freeze-dried keratin, and glutaraldehyde aqueous solution were added (Table S2). Subsequently, the solution was stirred successively at a fixed temperature (50 °C, 60 °C, 70 °C) for 4 h, 5 h, and 6 h). Afterwards, an appropriate amount of hydrochloric acid was added. After acidification for 3 h and neutral filtration, unreacted protein, aldehydes, and inorganic salts were washed off with excess deionized water. Finally, the grafted copolymers were dried at 40 °C for 48 h to obtain aminated lignin (AL). The design details of the proportion, time, temperature, and statistical analysis are shown in Supplementary Table S2.

### 3.4. Preparation of PVA/AL Composite Membrane

First, 5 wt% hybridization solution was prepared by adding PVA to deionized water. The PVA solution was heated at 95–100 °C for 2 h until the PVA was completely dissolved. Then, a certain amount of AL, dipotassium hydrogen phosphate, and deionized water were weighed and fully dissolved to obtain the AL solution. After mixing the PVA and AL solutions, 2 wt% of glycerol was added as a plasticizer, ultrasonicated for 1 h, and stirred for 2 h to ensure that the PVA and AL were evenly mixed. The mixture was poured into a polytetrafluoroethylene mold and first dried quickly at 100 °C for 1 h, then dried slowly at 60 °C for 12 h. Transparent films with a thickness of 110 (±10) μm were finally obtained. The concentrations of AL in the total solid content were 2.0, 5.0, 8.0, and 14 wt%, named PL-2, PL-5, PL-8, and PL-14, respectively.

### 3.5. Preparation of Coated Urea

Pellets were prepared using a laboratory-scale drum pelletizer (KARISHMA, Lab model coating pan, India). The urea granules were sieved to obtain spherical granules with a diameter of 0.3–0.4 cm and then coated. The rotational speed was set at 40 rpm, the heating fan power was set at 40 w, and the heating temperature was set to 70 °C. The urea particles were preheated for 10 min before coating, then the film liquid treatment was carried out to repeatedly and uniformly spray the urea prepared as described above onto the surface of the particles to obtain PVA-AL-coated urea. The coating process was carried out by alternating cycles of high-pressure spraying and hot air drying to avoid dissolution of the urea. This operation was repeated until the weight percentage of the dry film deposited on the outer surface of the urea reached 1, 2, and 3 wt%, respectively. After spraying was completed, all coated pellets were heated at 40 °C for 12 h and then sealed and set aside. The coating level was determined by the dry weight gain of the coated fertilizer, named UPL-1, UPL-2 and UPL-3, respectively. The coating ratio (η) was calculated as follows:

$$\eta (\%)=\frac{W-W_{0}}{W}\times 100\%$$

where *W* and *W*_0_ are the weights of the coated urea particles and the uncoated urea particles, respectively.

### 3.6. Characterization

Using a Fourier transform infrared spectrometer (iSTM 20 Thermo Fisher, Waltham, Massachusetts, USA) equipped with a Kinmen single attenuated total reflection accessory (ATR), the chemical structure of L, AL, and keratin hydrolysate PL coating material films was recorded in the range of 4000~600 cm^−1^ with a resolution of 16 cm^−1^. The elemental analysis of the L and AL was carried out using X-ray photoelectron spectroscopy (XPS) (madzu Corporation, Tokyo, Japan) and a PerkinElmer 2400II elemental analyzer (Perkin Elmer, Waltham, MA, USA). The thermal stabilities of the obtained films were determined using a thermogravimetric analyzer (TGA8000; PerkinElmer, Waltham, MA, USA) from room temperature (30 °C) to 500 °C in a nitrogen atmosphere at a rate of 10 °C min^−1^. The PL composites were characterized using a German Bruker D8 X-ray diffractometer (λ = 0.154 nm) in the range of 0–80° with a scanning rate of 10°/min.

### 3.7. Properties and Degradation of Membranes

#### 3.7.1. Mechanical Properties

The mechanical properties of the prepared films, i.e., the modulus of elasticity (Young’s modulus), tensile strength (σ), and elongation at break (ε), were calculated directly from the strain–stress curves. The tensile tests were performed using a benchtop universal/tensile testing machine (EZ-X Shimadzu) with a cross speed of 500 mm/min. The tests were carried out on dry rectangular specimens with a marking distance of 110 mm, width of 10 mm, and thickness of 110 ± 2 μm. The reported values are the average of five measurements.

#### 3.7.2. Water Absorption Rate

The water absorption rate of the resulting membranes was determined as described previously [27]. First, membrane samples (2 × 2 cm) were dried at 70 ± 3 °C to achieve a constant weight (*W*_1_). Second, the samples were immersed in distilled water for at least 24 h. The samples were then dried at 70 ± 3 °C to achieve a constant weight (*W*_1_). Finally, excess water was removed from the surface of the samples with filter paper and weighed as *W*_2_. Each sample was weighed at least three times to minimize experimental errors and the averaged value was obtained.

(1) $$\mathrm{Water}\;\mathrm{uptake}\;\%=\frac{W_{2}-W_{1}}{W_{1}}\times 100\%$$

#### 3.7.3. Ultraviolet Shielding Performance

A Shimadzu UV–visible spectrophotometer was used to determine the UV–visible absorption curve of the membrane. The sample was cut into a rectangle (4 cm × 1 cm) for measurement. According to the reference method [13], the transmittance was calculated by Equation (1), the UVA wavelength range was 320–400 nm, and the UVB wavelength range was 260–320 nm

(2) $$Average\;transmission\;(\%)=\sum_{Initial}^{End}T(\lambda )∕n$$

where *T*(λ) is the spectral transmittance of the sample, nm, λ is the wavelength, nm, and n is the absorbance number.

#### 3.7.4. Degradation of Membranes

Seven 4 × 4 cm PL-8 films were made using PTFE molds. The weight before placing it in the soil was noted as *W*_0_, and the weight (*W*_1_) was also obtained when the films were taken out at regular intervals (5, 10, 15, 20, 30, and 45), followed by washing and drying. The pH of the soil was approximately 8.2, the temperature was 25 °C, and the humidity was 47%. The degradation properties of the films were calculated as follows.

(3) $$\mathrm{degradation}\;\mathrm{rate}\;\%=\frac{W_{0}-W_{1}}{W_{0}}\times 100\%$$

### 3.8. Slow-Release Kinetics of Coated Urea Particles

Determination of urea nitrogen release from coated urea in soil was carried out by reference to the method in [48] with some modifications. The experiments were as follows: a polyvinyl alcohol (PVC) column was used, with a length of 45.0 cm and a diameter of 5.25 cm; a blue-capped flask was used to receive the filtrate under the column. The column was filled with 100 g of quartz sand as a substrate. Then, a mixture of sample (1 g) and soil (300.0 g) was placed and covered with 100 g of quartz sand as the top layer. The slow-release columns were stored at room temperature (27 ± 2 °C). To keep the soil at 30% moisture, 150 mL of water at a time was added to the PVC column. The filtrates from each slow-release column were collected after 1, 3, 5, 7, 10, 15, 20, and 30 days and stored at approximately 4 °C for further analysis by Kjeldahl. Untreated urea pellets were studied under the same conditions and served as a control. To evaluate the mechanism of urea release, a mathematical model was fitted to the data.

### 3.9. Potting Experiment

This experiment was conducted from 21 October 2023 to 21 November 2023. Each pot was filled with 300 g of soil. Soil physicochemical properties were nitrate nitrogen 12.56 mg/kg, ammonium nitrogen 9.31 mg/kg, rapidly available phosphorus 26.30 mg/kg, and rapidly available potassium 136.29 mg/kg. Five treatments were set: (1) nitrogen-free control (CK); (2) urea 0.5 g/pot (U); (3) 0.505 g UPL, 1% coated/pot (UPL-1); (4) 0.510 g containing 2% coating/pot (UPL-2); and (5) 0.515 g containing 3% coating/pot (UPL-3). The same amount of nitrogen was applied to each treatment and repeated three times. Potassium dihydrogen phosphate (0.5 g/pot) and potassium sulphate (0.6 g/pot) were applied as basal fertilizers according to local fertilization practices. All nitrogen fertilizers were applied as basal fertilizers, and the amount of fertilizer was doubled in the pot experiment. After harvesting, the dry weight, fresh weight, plant height, root length, and maximum leaf area were determined

## 4. Conclusions

In this study, AL-filled PVA-coated materials were prepared from high-purity alkaline lignin extracted from yak hair and aminated reagent polypeptides. An environmentally friendly urea-based slow-release fertilizer was designed and produced using rotary drum granulation and coating technology. The coating material was a biodegradable formulation made from aminated lignin (AL) and polyvinyl alcohol (PVA) composites with different AL contents (2%, 5%, 8%, and 14 wt%). Various characterizations showed good compatibility between AL and PVA, making for excellent characterizations of composites that are suitable for coating process. The slow release of the product in soil was simulated by soil column leaching experiments and the Ritger–Peppas model was suitable for describing the release rate of the encapsulated material, indicating that the UPL was released through both diffusion and solubilization. The potting test showed that UPL-2/UPL-3 significantly promoted the growth of *Brassica napus* plants. Therefore, the development of sustainable agroecological materials such as coated urea products has a wide range of application prospects for the sustainable development of modern agriculture.

## Figures and Tables

**Table 1:** Release experiment model fitting results.

Model	Fitting Equation	R^2^
Zero-level dynamics model	CR = 2.98293t + 18.06885	0.89030
First-order kinetic model	CR = 99.57789 × (1 − 2.71828^−0.08986t^)	0.97505
Second-order dynamic model	CR = 10.46328t ÷ (1 + 0.07808t)	0.98112
Higuchi model	CR = 17.99707t^1/2^ − 0.95934	0.99023
Ritger-Peppas model	CR = 17.02172 × (t^0.51495^)	0.99036
Polynomial fitting model	CR = 5.81572 − 0.09892t^2^ + 8.21041	0.97667

CR = (Mt/M1) represents the fraction of nutrients released at moment t, Mt is the cumulative release rate at moment t, and M1 is the total amount of urea released at the longest time of the experiment n is a diffusion index characterizing the release mechanism.

## Data Availability

All available data are contained within the article.

## Supplementary Materials

The following supporting information can be downloaded at: https://www.mdpi.com/article/10.3390/molecules29081699/s1.
