# Supplementary material for: Preparation and Application of Degradable Lignin/Poly (Vinyl Alcohol) Polymers as Urea Slow-Release Coating Materials
Source: Molecules. 2024 Apr 9;29(8):1699. doi: 10.3390/molecules29081699 (PMC11051779; doi:10.3390/molecules29081699)
Supplement: Supplementary file 1 [file molecules-29-01699-s001.zip › molecules-2940223-supplementary.pdf]

Supplementary Materials

Figures:

Figure S1. Base peak ion chromatogram from hydrolysate of yak keratin.

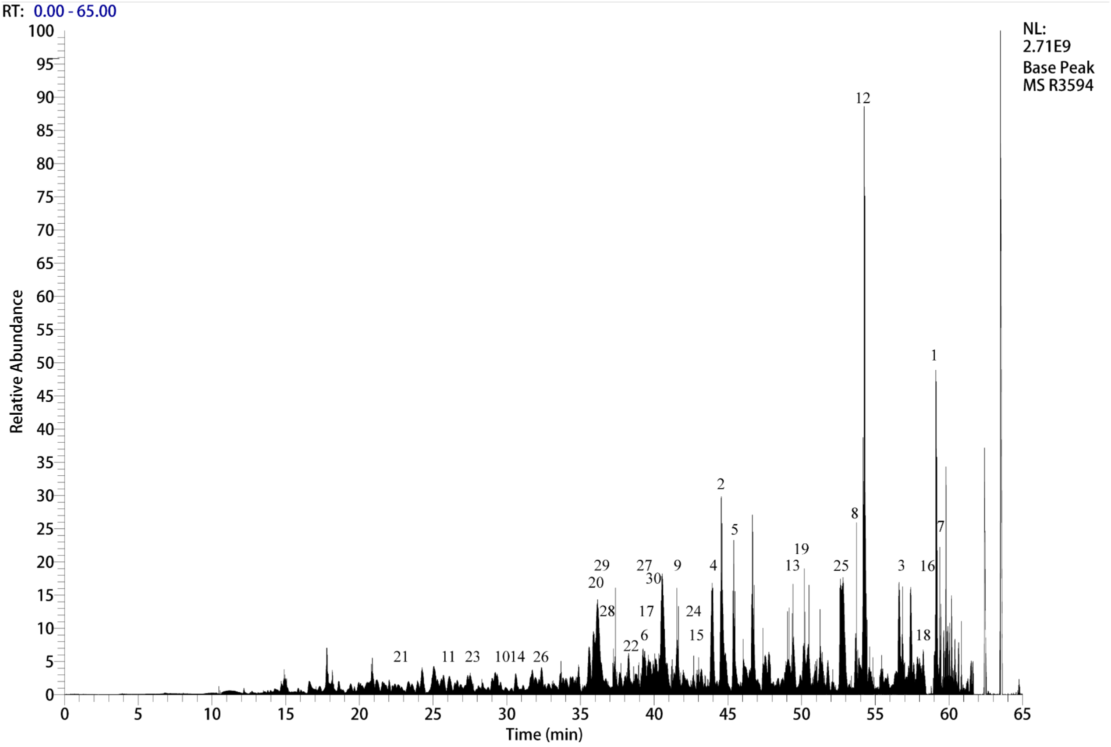

Highest abundance peptide (FLLD)

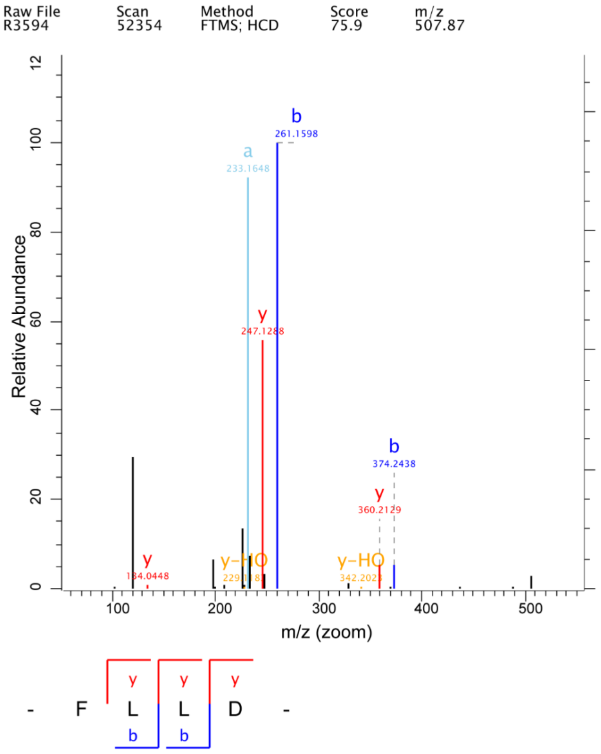

Tables:

Table S1. Peptides identified in the purified hydrolysis of the keratin samples.

No.	Mass	Charge	Peptide Sequences	Score	RT(min)
1	506.27405	1	FLLD	75.898	59.095
2	359.20564	1	LLD	69.294	44.538
3	506.27405	1	LFVE	51.96	57.168
4	416.2271	1	GLLD	75.898	43.872
5	373.22129	1	LLE	83.876	45.334
6	359.20564	1	LVE	83.876	39.785
7	375.21581	1	PLF	55.918	58.805
8	361.20016	1	FVP	25.656	53.489
9	416.2271	1	LGEV	70.862	41.078
10	487.3006	1	VKLE	70.863	29.299
11	416.2271	1	LEGV	31.275	25.161
12	416.2271	1	VEGL	21.483	54.697
13	361.20016	1	VPF	27.155	49.904
14	518.22241	1	EELE	119.89	30.603
15	423.20055	1	YLE	69.294	42.899
16	430.25801	1	WLL	41.83	59.101
17	529.3224	1	LRLE	62.086	39.193
18	375.21581	1	FPL	55.918	58.203
19	361.20016	1	PVF	41.83	50.213
20	372.27366	1	KLL	61.092	36.159
21	416.2271	1	EAVV	97.748	22.844
22	487.3006	1	KVLE	90	38.078
23	488.24823	1	LEVE	77.528	27.149
24	377.19507	1	YVP	55.918	42.577
25	1162.6234	2	EFVALKKDVD	187.5	53.379
26	529.3224	1	LERL	54.668	31.815
27	400.2798	1	LRL	53.534	38.928
28	358.22162	1	GLLG	62.263	36.153
29	377.19507	1	VPY	33.176	35.978
30	487.3006	1	LLKD	24.3	39.475

Table S2. The reaction condition and N content for the amination of L.

Group	Lignin (g)	Glutaraldehyde (ml)	Keratin (g)	Temperature (℃)	Time (h)	N%
AL1	1	1	2	40	4	2.35
AL2	1	1	4	50	5	4.68
AL3	1	1	6	60	6	5.14
AL4	1	2	2	50	6	4.16
AL5	1	2	4	60	4	5.22
AL6	1	2	6	40	5	6.17
AL7	1	3	2	60	5	3.78
AL8	1	3	4	40	6	5.17
AL9	1	3	6	50	4	6.78
K1		12.17	10.29	13.69	14.35	
K2		15.55	15.07	15.62	14.63	
K3		15.73	18.09	14.14	14.47	
k1		4.057	3.430	4.563	4.783	
k2		5.183	5.023	5.207	4.877	
k3		5.243	6.030	4.713	4.823	
R		1.187	2.600	0.643	0.0933	
Priority			B3>A3>C2>D2			
